# Grass Carp Reovirus VP35 Degrades MAVS Through the Autophagy Pathway to Inhibit Fish Interferon Production

**DOI:** 10.3389/fimmu.2021.613145

**Published:** 2021-03-23

**Authors:** Long-Feng Lu, Can Zhang, Zhuo-Cong Li, Xiao-Yu Zhou, Jing-Yu Jiang, Dandan Chen, Yong-An Zhang, Shun Li

**Affiliations:** ^1^Institute of Hydrobiology, Chinese Academy of Sciences, Wuhan, China; ^2^College of Advanced Agricultural Sciences, University of Chinese Academy of Sciences, Beijing, China; ^3^College of Fisheries and Life Science, Dalian Ocean University, Dalian, China; ^4^State Key Laboratory of Agricultural Microbiology, College of Fisheries, Huazhong Agricultural University, Wuhan, China

**Keywords:** VP35, GCRV, immune evasion, MAVS, interferon

## Abstract

Fish interferon (IFN) is a crucial cytokine for a host to resist external pathogens, conferring cells with antiviral capacity. Meanwhile, grass carp reovirus (GCRV) is a strong pathogen that causes high mortality in grass carp. Therefore, it is necessary to study the strategy used by GCRV to evade the cellular IFN response. In this study, we found that GCRV 35-kDa protein (VP35) inhibited the host IFN production by degrading mitochondrial antiviral signaling (MAVS) protein through the autophagy pathway. First, the overexpression of VP35 inhibited the IFN activation induced by polyinosinic-polycytidylic acid (poly I:C) and MAVS, and the expression of downstream IFN-stimulated genes (ISGs) was also decreased by using VP35 under the stimulation. Second, VP35 interacted with MAVS; the experiments of truncated mutants of MAVS demonstrated that the caspase recruitment domain (CARD) and proline-rich (PRO) domains of MAVS were not necessary for this binding. Then, MAVS was degraded by using VP35 in a dose-dependent manner, and 3-MA (the autophagy pathway inhibitor) significantly blocked the degradation, meaning that MAVS was degraded by using VP35 in the autophagy pathway. The result of MAVS degradation suggested that the antiviral capacity of MAVS was remarkably depressed when interrupted by VP35. Finally, in the host cells, VP35 reduced *ifn* transcription and made the cells vulnerable to virus infection. In conclusion, our results reveal that GCRV VP35 impairs the host IFN response by degrading MAVS through the autophagy pathway, supplying evidence of a fish virus immune evasion strategy.

## Highlights

- VP35 inhibits IFN activation.
- VP35 acts on the RLR signaling pathway by targeting MAVS.
- VP35 blocks the cellular IFN response and facilitates viral replication.

## Introduction

Grass carp reovirus (GCRV), a virus belonging to the family *Reoviridae*, causes serious outbreaks of grass carp hemorrhagic diseases and leads a very high mortality rate in grass carp (*Ctenopharyngodon idella*) (1). To date, three different subtypes of GCRV have been isolated: GCRV-I (e.g., GCRV-873), GCRV-II (e.g., GCRV-HZ08), and GCRV-III (e.g., GCRV-104) (2, 3). Among them, the most common strain is GCRV-II (4). The GCRV genome consists of 11 fragments named S1–S11, which are wrapped in a multilayer icosahedral capsid (5, 6). Comparing the protein sequences of the three genotypes of GCRV, it is found that the similarity is less than 20%, so the functions of the encoded proteins are also distinct. For example, the S11 segment of GCRV-I and GCRV-III is predicted to encode non-structured proteins (NS26 and VP8/VP15, respectively), while the S11 fragment in GCRV-II is predicted to encode a 35-kDa protein (VP35) with a conserved putative zinc-binding motif and acts as a putative outer-clamp protein (7–10). In recent years, more and more research studies are focused on the understanding of GCRV's involvement in pathogenesis and immune response (11). For instance, GCRV infection dramatically induces the transcription and expression of interferon (IFN) and IFN-stimulated genes (ISGs) (12). In addition, it has been shown that the expression levels of retinoic acid-inducible gene I (RIG-I) and melanoma differentiation-associated gene 5 (MDA5) genes in the spleen and liver are significantly upregulated after GCRV infection (13, 14). However, GCRV has been identified as an effective pathogen for grass carp, even host IFN successfully responded, means that there should be several strategies mediated by GCRV to avoid host IFN production, resulting in the inability to maximize the expression of IFN.

Upon infection with the virus, the first and fastest defense strategy for host cells is inducing IFN expression to establish an antiviral state (15). The produced IFNs bind to the IFN receptor on the cell membrane surface through autocrine or paracrine methods, and then the expression of a series of antiviral genes is activated (16, 17). In-depth research studies on signaling pathways in mammals reveal the mechanisms of IFN activation in the relatively early stages (18). In general, the cell membrane surface or intracellular pattern-recognition receptor (PRR) recognizes viral nucleic acids and then activates the corresponding downstream signaling pathways, which eventually facilitate the production of IFN (19). Among these pathways, the RIG-I-like receptor (RLR) signaling pathway plays an important role in this process (16). This specific process is as follows: an RLR family member (RIG-I or MDA5) senses viral RNA and also interacts with a mitochondrial antiviral signaling protein (MAVS) (20, 21). Then, the activated MAVS transmits signals to the downstream mediator of IFN regulatory factor 3 (IRF3) (MITA) and phosphokinase TRAF family member-associated NF-κB activator (TANK) binding kinase 1 (TBK1) (22). Subsequently, TBK1 phosphorylates IRF3/7, and the phosphorylated IRF3/7 translocates to the nucleus, binding to the promoter region of IFN or the related immune genes, ultimately promoting the establishment of an antiviral state (23, 24). To date, almost all the key molecules in the RLR signaling pathway and conserved signaling mechanisms have been successfully identified in fish (18). For instance, similar to mammals, zebrafish MAVS and MITA are key components of the RIG-I-mediated pathway to activates IFN expression and exhibit a powerful antiviral function (25). Moreover, crucian carp IRF3 is a typical ISG and induce IFN production efficiently (26).

As a pivotal molecule in the RLR signaling pathway, MAVS plays a vital role in resisting viral infection (27). MAVS contains an N-terminal caspase recruitment domain (CARD), a proline-rich (PRO) domain, and a C-terminal transmembrane (TM) domain (28). The overexpression of MAVS dramatically induces IFN and ISG expression while the depletion of MAVS abolishes host cellular antiviral function. The fish *mavs* gene is orthologous to mammalian *mavs*. It has been reported that fish MAVS also plays a crucial role in the induction of the antiviral immune response (29). Therefore, many viruses select MAVS as a target to escape host immune response (21, 30). For instance, Zika virus (ZIKV) non-structural protein 4A (NS4A) blocks the induction of IFN by dampening the RLR–MAVS interaction (28). Newcastle disease virus (NDV) V protein recruits E3 ubiquitin ligase RING-finger protein 5 (RNF5) and degrades MAVS through the ubiquitin–proteasome pathway resulting in the inhibition of IFN production (31).

To date, there is little information about the immune evasion mechanisms used by GCRV. Here, we reveal that the GCRV S11 segment-encoded VP35 interacts with MAVS and promotes its degradation through an autophagy pathway, thereby suppressing the host IFN production and permitting viral replication. These results provide an in-depth understanding of immune evasion mechanisms and the pathogenesis of GCRV.

## Materials and Methods

### Cells and Viruses

Dr. X. Liu (Institute of Hydrobiology, Chinese Academy of Sciences, Wuhan, Hubei Province) provided human embryonic kidney (HEK) 293T cells, which were maintained at 37°C in 5% CO_2_ in a Dulbecco's modified Eagle's medium (DMEM; Invitrogen, CA, USA) supplemented with 10% fetal bovine serum (FBS, Invitrogen, CA, USA). Grass carp ovary (GCO) cells and epithelioma papulosum cyprini (EPC) cells were obtained from China Center for Type Culture Collection (CCTCC) and were seeded at 28°C in 5% CO_2_ in medium 199 (Invitrogen, CA, USA) supplemented with 10% FBS.

Grass carp reovirus (106 strains, GCRV-II) was given by Zeng Lingbing (Yangtze River Fisheries Research Institute, Chinese Academy of Fishery Sciences, Hubei, China). Since GCRV-II cannot cause cytopathic effect (CPE), but can reproduce in GCO cells, the culture medium of GCO cells infected with GCRV-II for 8 days was collected and stored at −80°C until use. Spring viremia of carp virus (SVCV) was propagated in the EPC cells until CPE was complete, and the culture medium was harvested and stored at 28°C until needed.

### Plasmid Construction and Reagents

The open reading frames (ORFs) of VP35 (KC201176.1), grass carp RIG-I (JX649222.1), MAVS (KF366908.1), TBK1 (JN704345.1), and IRF3 (KT347289.1) were amplified by using a PCR and then cloned into pcDNA3.1(+), pCMV-Myc, pCMV-HA, and pCMV-Tag2C vectors. For the cell location experiment, the ORF of VP35 was inserted into the pEGFP-N3 vector (Clontech, CA, USA). The ORFs of MAVS, TBK1, and IRF3 were also cloned into the pCS2-mCherry vector (Clontech, CA, USA). Insertion of the corresponding 5' flanking regulatory region of an IFN1 promoter (GU139255.1) into a pGL3-Basic luciferase reporter vector (Promega, Madison, WI, USA) to generate an IFN1pro-Luc construct for a promoter activity analysis. The interferon-stimulated response element-(ISRE)-Luc plasmid in pGL3-Basic Luciferase Reporter Vector (Promega, Madison, WI, USA) and DrIFNϕ1pro-Luc were described in a previous study (32). All constructed expression vectors were verified by DNA sequencing.

### Luciferase Activity Assay

The EPC cells were seeded into 24-well plates (~2 × 10^4^ cells) overnight and transfected together with various vectors: MAVS/TBK1/MITA/IRF3/IRF7, IFN1pro/DrIFNϕ1pro/ISRE-Luc, and pRL-TK at a ratio of 10:10:1. The empty vector [pcDNA3.1(+)] was used to ensure an equal amount of DNA per well. In 24-h post-transfection, cells were transfected with 1μg/ml polyinosinic-polycytidylic acid (poly I:C) (Sigma-Aldrich, MO, USA, P1530) was conducted by using FishTrans (MeiSenTe Biotechnology, Beijing, China). After another 24 h, cells were washed with phosphate-buffered saline (PBS) and then lysed for detecting luciferase activity by a dual-luciferase reporter gene analysis system according to the instructions (Promega, Madison, WI, USA).

### Transient Transfection and Virus Infection

Transient transfection of the EPC cells grown in 6-well (~1.5 × 10^5^ cells) or 24-well plates (~2 × 10^4^ cells) was observed by using FishTransDNA Transfection Reagent according to the instructions of the manufacturer. Antivirus assays were performed with the EPC cells seeded in 24-well plates. GCRV-II, which could not cause CPE through the viral RNA, propagates successfully. In order to observe a significant antiviral effect and determine the virus titer, another RNA virus SVCV is chosen in this study. Each well was transfected with 0.5 μg of empty vector or pcDNA3.1 (+)-VP35. After 24 h, cells were infected with SVCV [multiplicity of infection (MOI) = 0.001]. After 2 or 3 days, supernatant liquor was collected to detect virus titers, and then monolayer cells were fixed with 4% paraformaldehyde (PFA) and stained with 1% crystal violet to observe CPE. Subsequently, 200 μl of culture medium was collected after 48 h of infection and used for the detection of virus titers according to the method of Reed and Muench. The supernatants were serially diluted 3-fold, and then 100 μl was added to a monolayer of EPC cells cultured in a 96-well plate (~ 3 × 10^3^ cells). After 48 or 72 h, the medium was removed and washed with PBS, fixed with 4% PFA, and stained with 1% crystal violet. Viral titer was expressed as 50% tissue culture infectious dose (TCID_50_/ml).

### RNA Extraction, Reverse Transcription, and Quantitative Real-Time PCR

Total RNAs were extracted by a Trizol reagent (Invitrogen, CA, USA). Genomic DNA was thoroughly digested by RNase free DNase (Promega Madison, WI, USA). Using a GoScript reverse transcription system, complementary DNA (cDNA) is synthesized according to the instructions of Promega. Quantitative real-time PCR (qPCR) was performed on the CFX96 real-time system (Bio-Rad) by using Fast SYBR Green PCR Master Mix (Bio-Rad). The PCR procedure is as follows: 95°C for 5 min, and then 40 cycles: 95°C for 20 s, 60°C for 20 s, and 72°C for 20 s. The internal control is *β-actin* gene. The 2^−ΔΔCt^ method was used to calculate the relative fold changes in contrast to the corresponding controls. The qPCR primers (designed by the software Primer Premier 5) used in this study were listed in Table 1.

**Table 1:** Primers used in this study.

**Name**	**Sequence (5^′^ → 3^′^)**	**Purpose**
*svcv-n-*FP	TGAGTGCTGAGGACGAT	qPCR
*svcv-n-*RP	TTTGTGAGTTGCCGTTA	
*svcv-p-F*P	TTGGACCTGGGATAGTGA	
*svcv-p-*RP	CTTGCTTGGTTTGTGGG	
*svcv-g-*FP	CGACCTGGATTAGACTTG	
*svcv-g-*RP	AATGTTCCGTTTCTCACT	
*svcv-l-*FP	GCCCACTTTGCATCCAGTCC	
*svcv-l-*RP	GAGATGCCACAGACTCCTCC	
*svcv-m-*FP	TACTCCTCCCACTTACGA	
*svcv-m-*RP	CAAGAGTCCGAGAAGGTC	
*isg15-*FP	CCCCTTTCCAAGTGTTCGTC	
*isg15-*RP	ATGGTGCTTCCAGATGTGATGT	
*vig1-*FP	AGCGAGGCTTACGACTTCTG	
*vig1-*RP	GCACCAACTCTCCCAGAAAA	
*ifn-*FP	ATGAAAACTCAAATGTGGACGTA	
*ifn-*RP	GATAGTTTCCACCCATTTCCTTAA	
*irf7-*FP	GGAGGACCAACACAAAGTCTATC	
*irf7-*RP	CATTTCCTCCACTTGGCTGAG	
*rig i-*FP	TGCTGGACCGGATGTGTTATCT	
*rig i-*RP	TGGTGATCGATGGTTCGATTCT	
*S11-*FP	TGTCAATTCCACCACCCC	
*S11-R*P	TTCAGATTCACTATTCCCTCCA	
*β-actin-*FP	CACTGTGCCCATCTACGAG	
*β-actin-*RP	CCATCTCCTGCTCGAAGTC	

### Co-immunoprecipitation Assay

In a co-immunoprecipitation (Co-IP) experiment, HEK 293T cells are used instead of the EPC cells due to their higher transfection efficiency. HEK 293T cells are inoculated in 10-cm^2^ culture dishes (~ 6 × 10^6^ cells) overnight, and co-transfected 10 μg plasmids (as shown in the figures). After 24 h, the culture medium was discarded and the cell monolayer was washed with PBS, then the cells were lysed in 1.2-ml cell lysates at 4°C for 1 h on a rocker platform. The compositions of lysate are as follows: 1% Nonidet P-40, 50 mM Tris-HCl (pH 7.4), 150 mM NaCl, 1 mM EDTA, 1 mM NaF, 1 mM sodium orthovanadate [Na_3_VO_4_], 1 mM phenylmethylsulfonyl fluoride (PMSF), 0.25% sodium deoxycholate, and protease inhibitor mixture (Sigma-Aldrich, MO, USA). Then the lysate is transferred to a 2-ml centrifuge tube and centrifuge at 12,000 × *g* for 15 min at 4°C. Finally, the supernatant is transferred to a new 1.5-ml tube and mixed with 20-μl anti-Myc/Flag affinity gel (Sigma-Aldrich, MO, USA) overnight at 4°C with constant agitation. Immunoprecipitated proteins were collected by centrifugation at 5,000 × *g* for 1 min at 4°C, washed three times with lysis buffer, and finally resuspended in 100 μl 2 × sodium dodecyl sulfate (SDS) sample buffer. Immunoprecipitation and whole-cell lysates (WCLs) are analyzed by immunoblotting (IB) with the designated antibodies (Abs).

### Western Blotting

Immunoprecipitation or WCLs were separated by 10 or 15% SDS-polyacrylamide gel electrophoresis (PAGE) and then transferred to a polyvinylidene fluoride (PVDF) membrane (Bio-Rad) by using a semidry transfer method. The membranes were blocked in a tris-buffered saline (TBST) buffer (25 mM Tris-HCl, 150 mM NaCl, 0.1% Tween 20, pH 7.5) containing 5% skimmed milk powder for 1 h at room temperature, incubated overnight at 4°C with primary Abs diluted in an appropriate ratio, and washed three times with TBST for 5 min each. Then, the membranes were incubated with the second Abs for 1 h at room temperature. After washings three times, the membranes were stained with the Immobilon Western chemiluminescent horseradish peroxidase (HRP) substrate (Millipore), and the used Image Quant LAS 4000 system (GE Healthcare, IL, USA) for the detection. The dilution ratios of Abs were as follows: anti-β-actin (Cell Signaling Technology, MA, USA) (1:2,000), anti-Flag/HA (Sigma-Aldrich, MO, USA) (1:3,000), anti-Myc (Santa Cruz Biotechnology, TX, USA) (1:3,000), and HRP-conjugated anti-rabbit IgG or anti-mouse IgG (Thermo Scientific, MA, USA) (1:5,000).

### Fluorescence Microscope

The EPC cells inoculated on the coverslip in the six-well plates (~ 6 × 10^4^ cells) and transfected with the relevant plasmids are shown in the figures. After 24 h, the cells were washed two times with PBS and were fixed with 4% PFA for 1 h. After washing the cells three times with PBS, the cells were stained with 4',6-diphenyl-2-phenylindole (DAPI) (1 μg/ml; Beyotime, Jiangsu, China) for 15 min at room temperature in dark. Finally, the coverslips were washed and observed using a confocal microscope under a × 63 oil immersion objective lens (SP8; Leica, Wetzlar, Germany).

### Statistical Analysis

Luciferase, qPCR, and virus titer detection data are expressed as mean ± SEM (*n* ≥ 3). The values of *p* were calculated by using the Student's *t*-test or one-way ANOVA with a Dunnett's *post-hoc* test (SPSS Statistics, version 19; IBM, NY, USA). A value of *p* < 0.05 was considered to be statistically significant.

## Results

### Grass Carp Reovirus VP35 Inhibits Poly I:C or SVCV-Induced Interferon Expression

**Figure 1:**
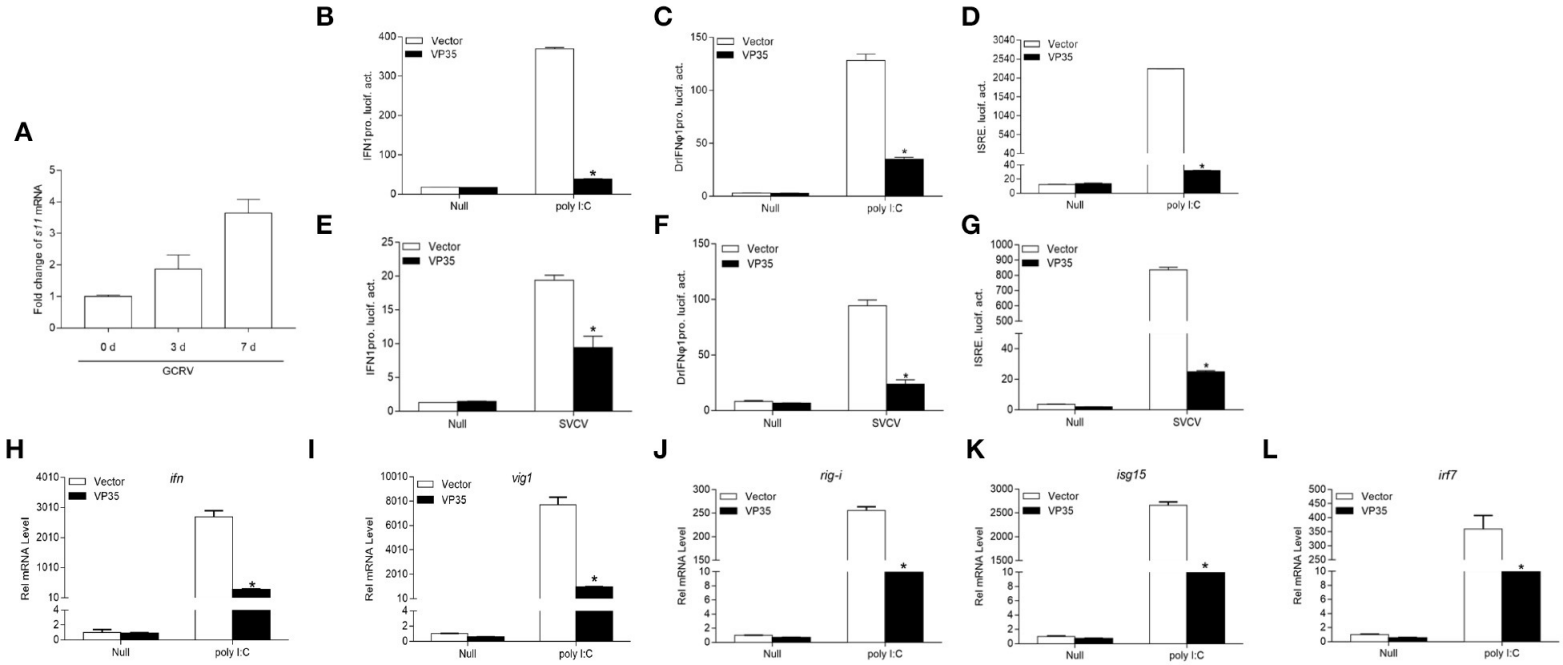
Grass carp reovirus (GCRV) 35-kDa protein (VP35) inhibits polyinosinic-polycytidylic acid (poly I:C) or spring viremia of carp virus- (SVCV-) induced interferon (IFN) expression. **(A)** Quantitative real-time PCR (qPCR) detects the transcription level of s11 after grass carp ovary (GCO) infection. GCO cells were seeded on six-well plates overnight and infected with GCRV [100 μl of the filtered virus containing supernatant of frozen and thawed GCO cells, which was diluted 100 times with phosphate buffered saline (PBS)]. At 1, 3, and 7 day post-infection (dpi), total RNAs were extracted for qRCR detection. **(B–G)** Overexpression of VP35 inhibits poly I: C or SVCV-induced IFN1pro, DrIFNϕ1pro, and interferon-stimulated response element (ISRE) **(D,G)** activation. Inoculated GCO cells into a 24-well plate overnight, and transfected with 250 ng IFN1pro-Luc **(B,E)**, DrIFNϕ1pro **(C,F)**, or ISRE-Luc **(D,G)** and 25 ng pRL-TK, plus 250 ng VP35-pcDNA3.1 (+), or pcDNA3.1 (+) (control vector). After 24 h, the cells were untreated or treated with SVCV [multiplicity of infection (MOI) = 1] or poly I:C (1 μg/ml). The cells were collected and then lysed for luciferase assay. **(H–L)** Overexpression of VP35 inhibits the expression of *ifn* and other IFN-stimulated genes (ISGs) induced by poly I:C. The EPC cells seeded in six-well plates overnight were transfected with 2 μg VP35-pcDNA3.1 or empty vector and transfected with poly I:C at 24 h post-transfection. At 24 h after stimulation, total RNAs were extracted to examine the mRNA levels of cellular *ifn* **(H)**, *vig1* **(I)**, *rig-i* **(J)**, *isg15* **(K)**, and *irf7* **(L)**. The relative transcriptional levels were normalized to the transcription of *β-actin* and represented as fold induction relative to the transcriptional level in the control cells, which was set to 1. Data are expressed as mean ± SEM, *n* = 3. Asterisks indicate a significant difference from the control (*p < 0.05).

Although grass carp possesses a mature immune system to resist the invasion of viruses, GCRV causes large-scale deaths. We speculated that functional viral protein(s) mediate the GCRV immune evasion. After screening the GCRV segments-encoded proteins, we found that S11 segment-encoded VP35 has the potential to play a crucial role in the battle with host. After infecting GCO cells with GCRV, the viral mRNA increased from day 3 until day 7, meaning that GCRV replicated successfully in GCO cells (Figure 1A). The host IFN response is the first and most pivotal line of defense against virus infection; therefore, GCRV VP35 was checked in the subsequent luciferase assay on IFN modulation. First, the promoter of grass carp IFN1 (IFN1pro) was remarkably activated by poly I:C; however, VP35 severely inhibited this induction (Figure 1B). To explore the extensive capacity of VP35 on IFN expression, zebrafish IFN promoter (DrIFNϕ1pro) and ISRE activities were also monitored, and a strong decline was also observed in these two groups when VP35 was present (Figures 1C,D). Subsequently, the function of VP35 negatively regulated IFN promoters was verified under virus infection, and the induction of these three promoters by SVCV was antagonized by VP35 (Figures 1E–G). These data suggest that VP35 inhibits the host IFN production under an exogenous stimulation. Moreover, the expressions of ISGs were monitored at the mRNA level. Besides the expression of *ifn*, several ISGs were depressed in the VP35 group, including *vig1, isg15-1, rig-i*, etc. (Figures 1H–L) (33). These results suggest that the host IFN response is significantly suppressed by GCRV VP35.

### VP35 Suppresses the Induction of Interferon Using Targeting MAVS

**Figure 2:**
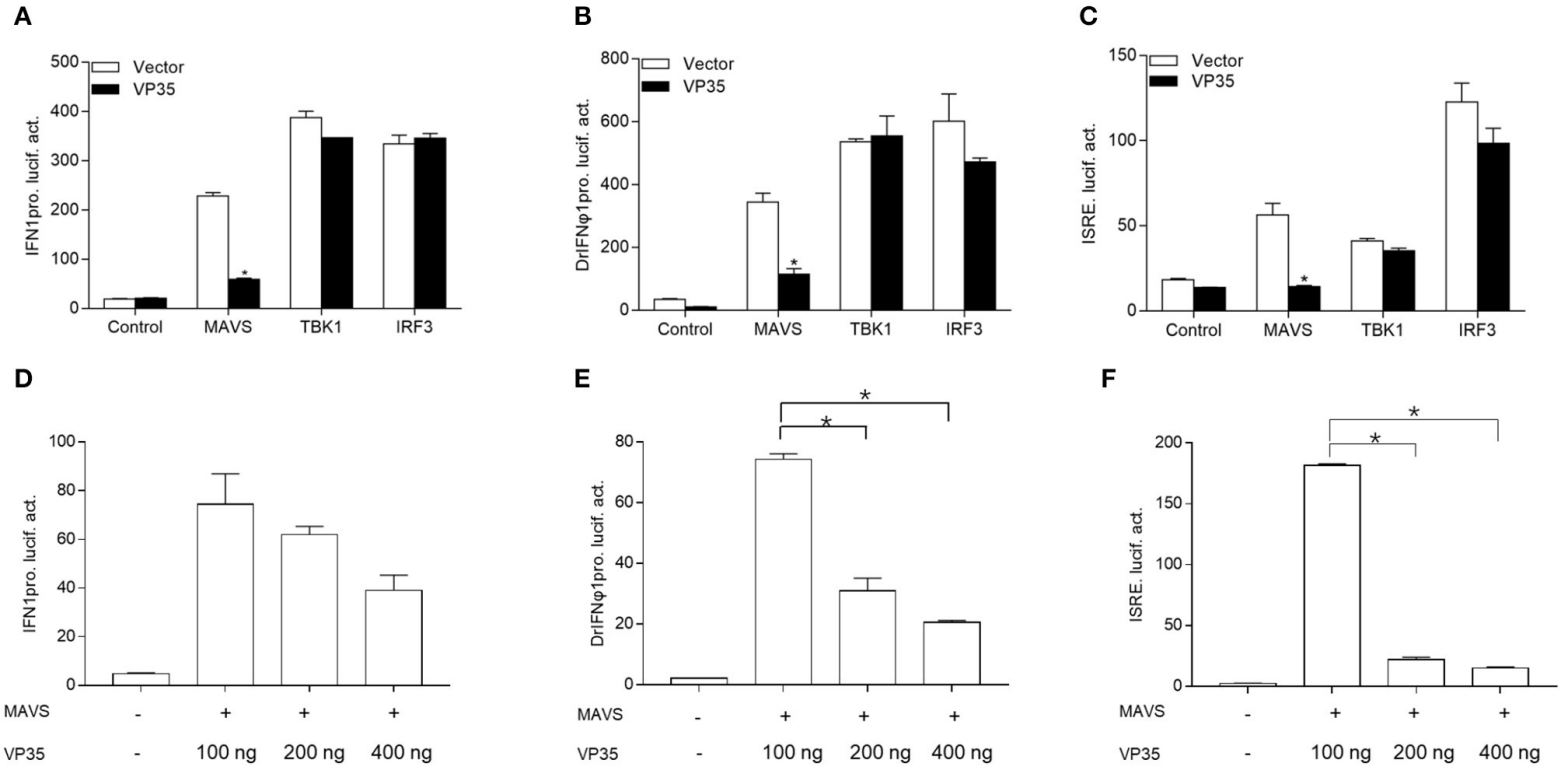
VP35 suppresses MAVS-activated IFN induction. **(A–C)** GCO cells were seeded into a 24-well plate and co-transfected with 250 ng expression plasmids MAVS/TRAF family member-associated NF-κB activator (TANK) binding kinase 1 (TBK1)/IFN regulatory factor 3 (IRF3) with 250 ng VP35-pcDNA3.1(+) or empty vector, plus 250 ng IFN1pro-Luc **(A)**, DrIFNϕ1pro-Luc **(B)**, and ISRE-Luc **(C)**, and 25 ng pRL-TK. After 24 h, the cells were lysed for monitoring luciferase activity. **(D–F)** Overexpression of VP35 inhibited the activities of IFN1pro, DrIFNϕ1pro and ISRE induced by MAVS in a dose-dependent manner. Inoculated GCO cells in a 24-well plate overnight, and co-transfected with 250 ng IFN1pro-Luc **(D)**, DrIFNϕ1pro-Luc **(E)**, and ISRE-Luc **(F)**, 25 ng pRL-TK, 250 ng MAVS and VP35-pcDNA3.1 (+) (0, 100, 200, or 400 ng). Data are expressed as mean ± SEM, *n* = 3. Data are expressed as mean ± SEM, *n* = 3. Asterisks indicate significant differences from control (*p < 0.05).

It has been reported that the fish RLR signaling pathway plays a vital role in the production of IFN (18, 34). It is unknown whether GCRV VP35 inhibits IFN expression through regulating this process; thus, the grass carp RLR key molecular constructs and IFN promoter were used to confirm this. As shown in Figure 2A, the RLR cascades significantly induced the activity of the grass carp IFN1 promoter, and the overexpression of VP35 dampened MAVS-induced IFN1pro activity dramatically but did not affect the activation of IFN1 prostimulated by TBK1 and IRF3. Moreover, VP35 had a dose-dependent effect on MAVS-mediated IFN1 proactivation (Figure 2D). In addition, DrIFNϕ1pro and ISRE were used for the detection. Similarly, VP35 blocked the transcription of IFN induced by MAVS but had no obvious effect on the IFN activity induced by other key molecules (Figures 2B,C). In addition, this suppression was dose-dependent (Figures 2E,F). Collectively, these data demonstrate that VP35 represses the production of IFN likely *via* targeting MAVS in the RLR signaling pathway.

### VP35 Is Distributed Throughout the Cells and Interacts With RLR Molecules

**Figure 3:**
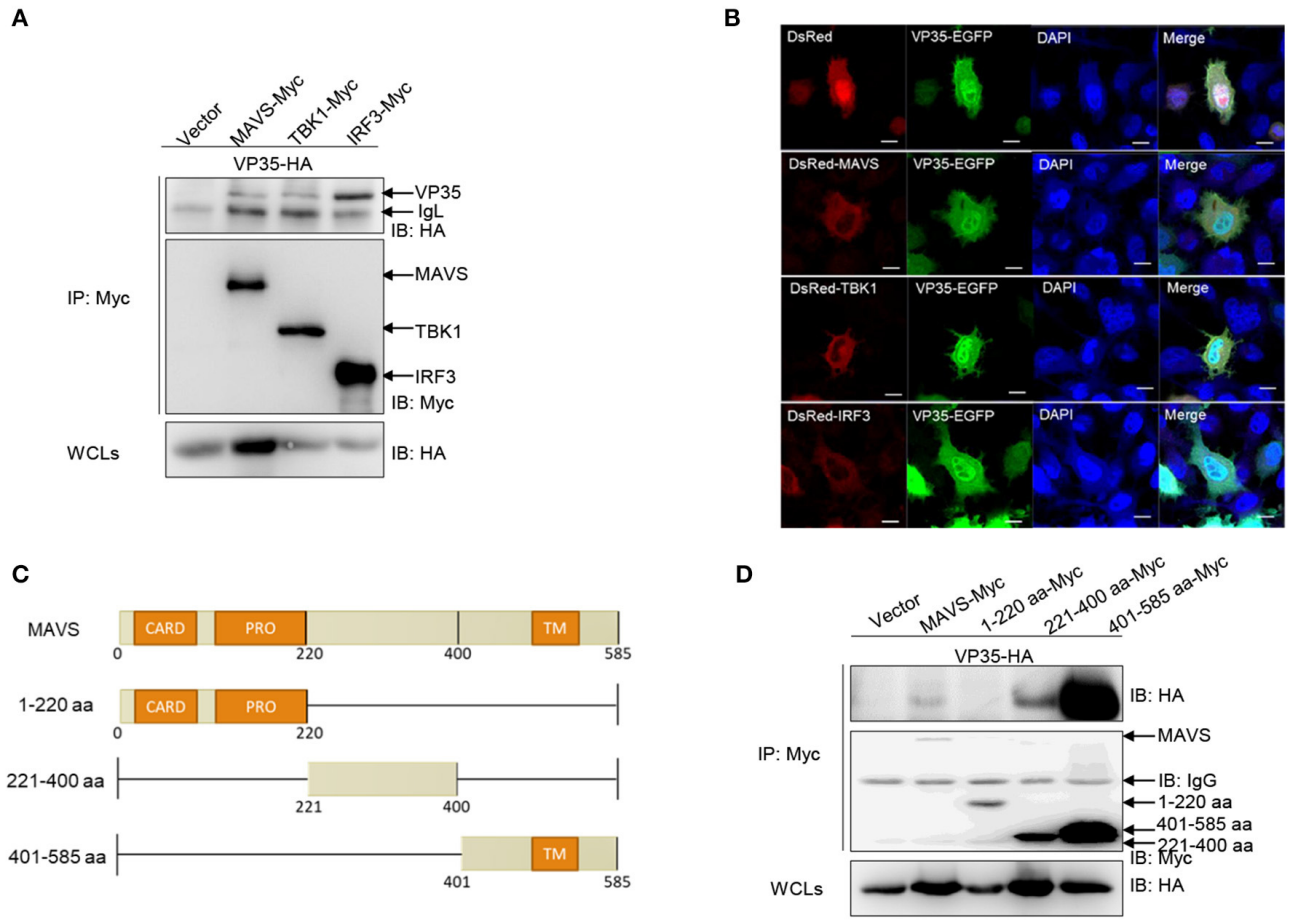
VP35 is distributed throughout the cells and interacts with (RIG-I)-like receptor (RLR) molecules. **(A)** Inoculated human embryonic kidney (HEK) 293T cells into 10-cm^2^ dishes and transfected with the indicated plasmids (5 μg each). After 24 h, the cells were lysed and immunoprecipitated (IP) with Anti-Flag Affinity Gel. Then the immunoprecipitate and whole-cell lysates (WCLs) were analyzed by immunoblotting (IB) with anti-Flag and anti-Myc antibodies (Abs), respectively. **(B)** EPC cells were seeded into a six-well plate, and transfected with 1 μg MAVS-DsRed, TBK1-DsRed, and IRF3-DsRed and 1 μg VP35-EGFP or empty vector. After 24 h, the cells were fixed and analyzed by confocal microscopy. The green signal indicates the over-expressed VP35 protein, the red signal indicates the over-expressed MAVS, TBK1, and IRF3, and the blue indicates the nuclear area (original magnification 63 ×; oil immersion objective). Scale bar, 10 μm. **(C)** Schematic representation of full-length MAVS and its mutants. **(D)** The 221–400 aa and 401–585 aa of MAVS are responsible for its interaction with VP35. The experiments were performed similarly as described above for **(A)**. All experiments were repeated at least three times with similar results.

Given that VP35 blocks IFN expression activated by RLR molecules, an investigation is needed whether VP35 associates with RLRs at the protein level. First, a Co-IP assay was performed. After co-transfecting VP35-HA and MAVS/TBK1/IRF3 with Myc tag in 293T cells, anti-Myc Ab-immunoprecipitated protein complexes containing MAVS, TBK1, and IRF3 were recognized by the anti-HA Ab, meaning that VP35 interacted with MAVS, TBK1, and IRF3 (Figure 3A). Next, the associations of VP35 and RLR molecules were confirmed by a subcellular localization analysis. As shown in Figure 3B, the VP35-EGFP signals were distributed throughout the cells. Moreover, the red signals of MAVS/TBK1/IRF3 were observed in the cytoplasm, which partially overlapped the distribution of VP35. Then, which domain of MAVS was necessary for the interaction with VP35 was determined. We constructed a series of MAVS mutants: 1–220 aa (including N-terminal CARD and PRO domains), 221–400 aa, and 401–585 aa (including C-terminal TM domain). The schematics of the full-length MAVS and its truncations were shown in Figure 3C. The results showed that full-length MAVS-Myc as well as the mutants MAVS (221–400 aa) and MAVS (401–585 aa) interacted with VP35 but not MAVS (1–220 aa), indicating that the CARD and PRO domains of MAVS were not necessary for its binding to VP35 (Figure 3D). These data suggest that VP35 distributes throughout the cells and interacts with MAVS of the N-terminal CARD and PRO domains.

### VP35 Degrades MAVS *via* Autophagy Pathway

**Figure 4:**
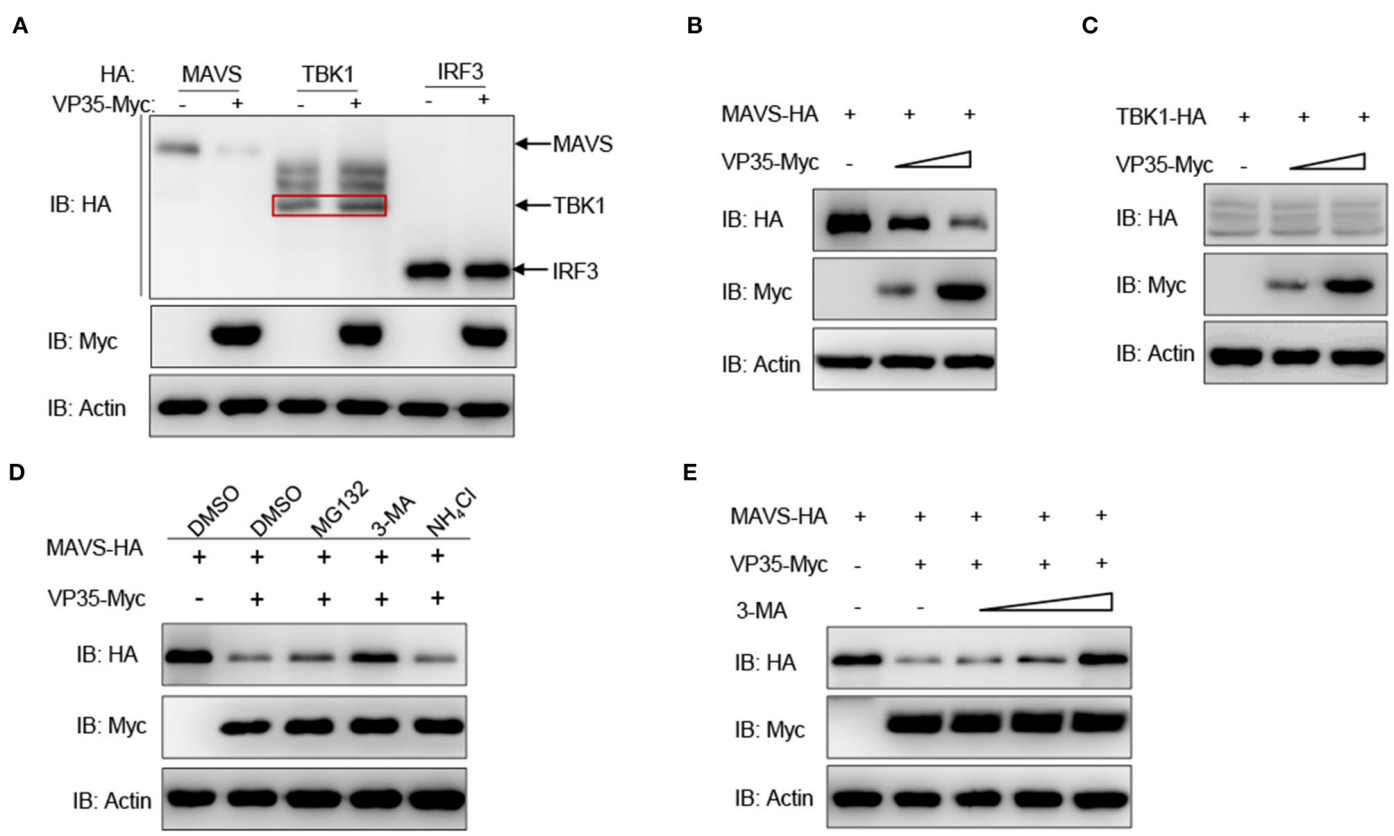
VP35 degrades MAVS through the autophagy pathway. **(A)** EPC cells were seeded in six-well plates overnight and co-transfected with 1 μg of MAVS/TBK1/IRF3-HA and 1 μg of empty vector or VP35-Myc for 24 h. The WCLs were subjected to IB with the anti-HA, anti-Myc, and anti-β-actin Abs. **(B,C)** EPC cells were seeded into six-well plates overnight and co-transfected with 1 μg MAVS-HA **(B)** or TBK1-HA **(C)** plus various concentration of VP35-Myc (0, 1, or 2 μg, empty vector was used to make up the rest). After 24 h, the WCLs were subjected to IB with anti-Myc, anti-HA, and anti-β-actin Abs. **(D)** Effects of inhibitors on VP35-mediated degradation of MAVS. EPC cells were seeded in six-well plates overnight and co-transfected with 1 μg MAVS-HA and 1 μg VP35-Myc. At 18 h post-transfection, the cells were treated with the dimethyl sulfoxide (DMSO), MG132 (20 μM), 3-MA (2 mM), or NH_4_Cl (20 mM) for 6 h prior to being harvested for IB analysis of WCLs with the anti-HA, anti-Myc, and anti-β-actin Abs. **(E)** VP35-induced MAVS degradation is rescued by 3-MA in a dose-dependent manner. EPC cells were seeded in six-well plates overnight and co-transfected the indicated plasmids. At 18 h post-transfection, the cells were treated with DMSO or 3-MA (1, 2, or 4 mM) for 6 h. Then, the cells were harvested for IB with the Abs indicated. All experiments were repeated at least three times with similar results.

To gain further insight into the detailed regulatory mechanism of VP35 on MAVS, the effect of VP35 on the RLR axis at the protein level was determined. As shown in Figure 4A, the overexpression of VP35 clearly decreased the abundance of MAVS while it did not influence the expression of TBK1 or IRF3. Furthermore, the decline of MAVS was intensified significantly with the increasing doses of VP35 (Figure 4B). In contrast, the amount of TBK1 did not change with the different concentrations of VP35 (Figure 4C). Overall, three main protein degradation systems exist in organisms: the autophagy pathway (corresponding inhibitor: 3-MA), ubiquitin–proteasome pathway (corresponding inhibitor: MG132), and lysosomal pathway (corresponding inhibitor: NH_4_Cl). To elucidate the specific degradation manner for MAVS, the cells were treated with the corresponding inhibitors. The VP35-mediated degradation of MAVS was blocked by the autophagy inhibitor 3-MA but not influenced by NH_4_Cl or MG132 (Figure 4D). Moreover, MAVS protein levels were gradually enhanced with increasing doses of 3-MA, meaning that VP35 degrades MAVS in an autophagy manner (Figure 4E). In summary, these results suggest that VP35 induces the degradation of MAVS through the autophagy pathway.

### VP35 Affects MAVS-Mediated Signal Transmission and Host Interferon Production

**Figure 5:**
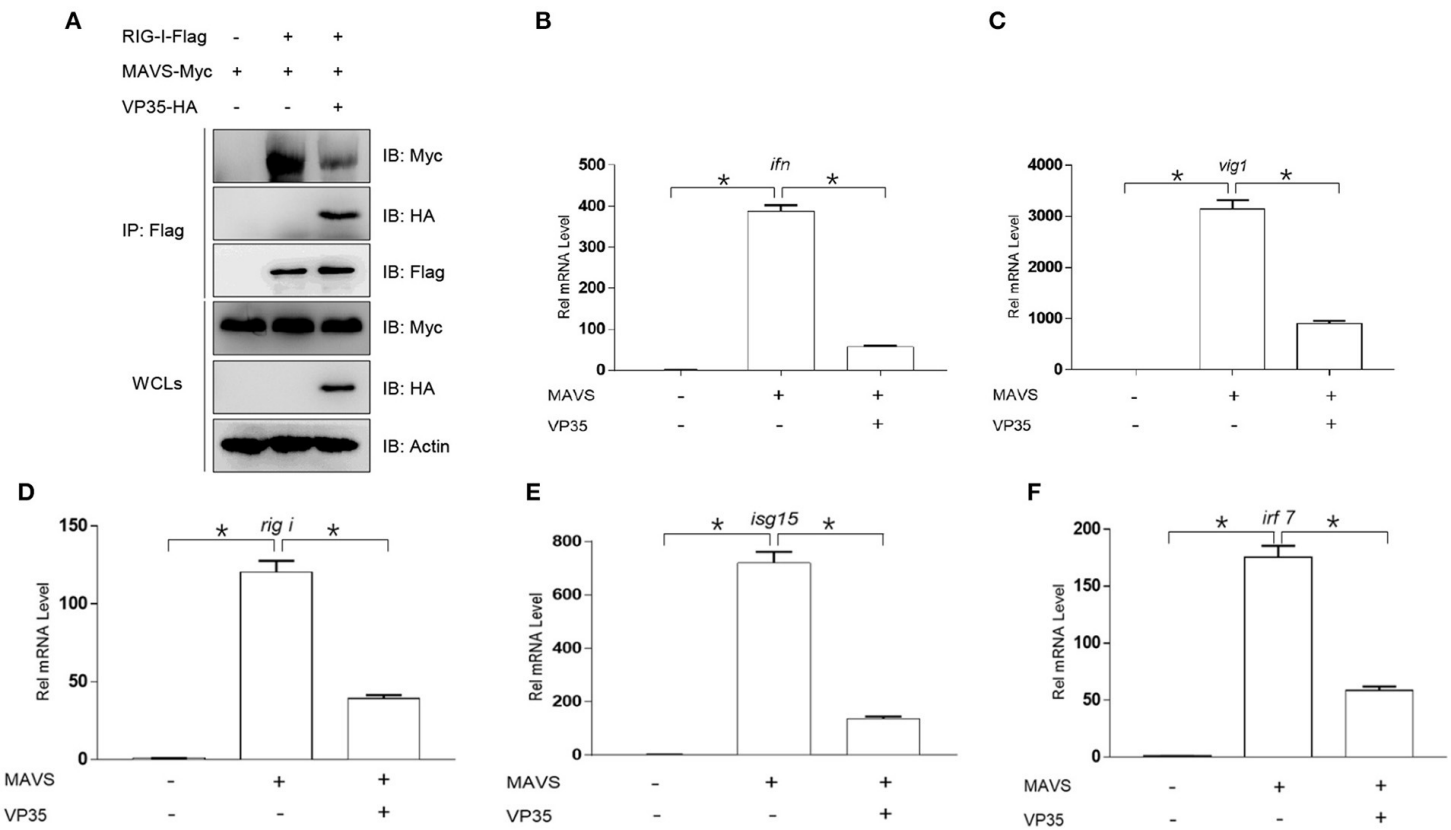
VP35 affects MAVS-mediated signal transmission and the host IFN production. **(A)** HEK 293T was seeded in 10-cm^2^ dishes and transfected with 4 μg RIG-I-Flag, 4 μg MAVS-Myc, and 4 μg VP35-HA. After 24 h, the cells were lysed for co-immunoprecipitation (Co-IP) with Anti-Flag Affinity Gel. Then the immunoprecipitate and WCLs were analyzed by IB with anti-Flag, anti-HA, anti-Myc, and anti-β-actin Abs. **(B–F)** EPC cells were seeded in six-well plates and transfected with 2 μg MAVS-Myc and VP35-HA, after 24 h, total RNAs were extracted to examine the transcriptional levels of *ifn/vig1/rig-i/isg15/irf7*. The relative transcriptional levels were normalized to the transcriptional level of the *β-actin* gene and were represented as fold induction relative to the transcriptional level in the control cells, which was set to 1. Data are expressed as mean ± SEM, *n* = 3. Asterisks indicate significant differences from control values (*p < 0.05).

It has been reported that MAVS interacts with the CARDs of RIG-I *via* its CARD, resulting in the activation of MAVS, which induces IFN expression (35). This interaction also exists in zebrafish (36). Thus, we speculated that VP35 affected the association between MAVS and RIG-I. As shown in Figure 5A, MAVS was associated with RIG-I, and VP35 dramatically weakened this interaction. Next, we investigated whether VP35 regulated MAVS-induced IFN expression. In qPCR assays, the overexpression of VP35 repressed MAVS-activated expression of *ifn* and other ISG mRNAs (Figures 5B–F). Taken together, these data suggest that VP35 attenuates the MAVS-induced IFN production by weakening the RIG-I/MAVS interaction.

### VP35 Blocks MAVS-Induced Cellular Antiviral Immune Response

**Figure 6:**
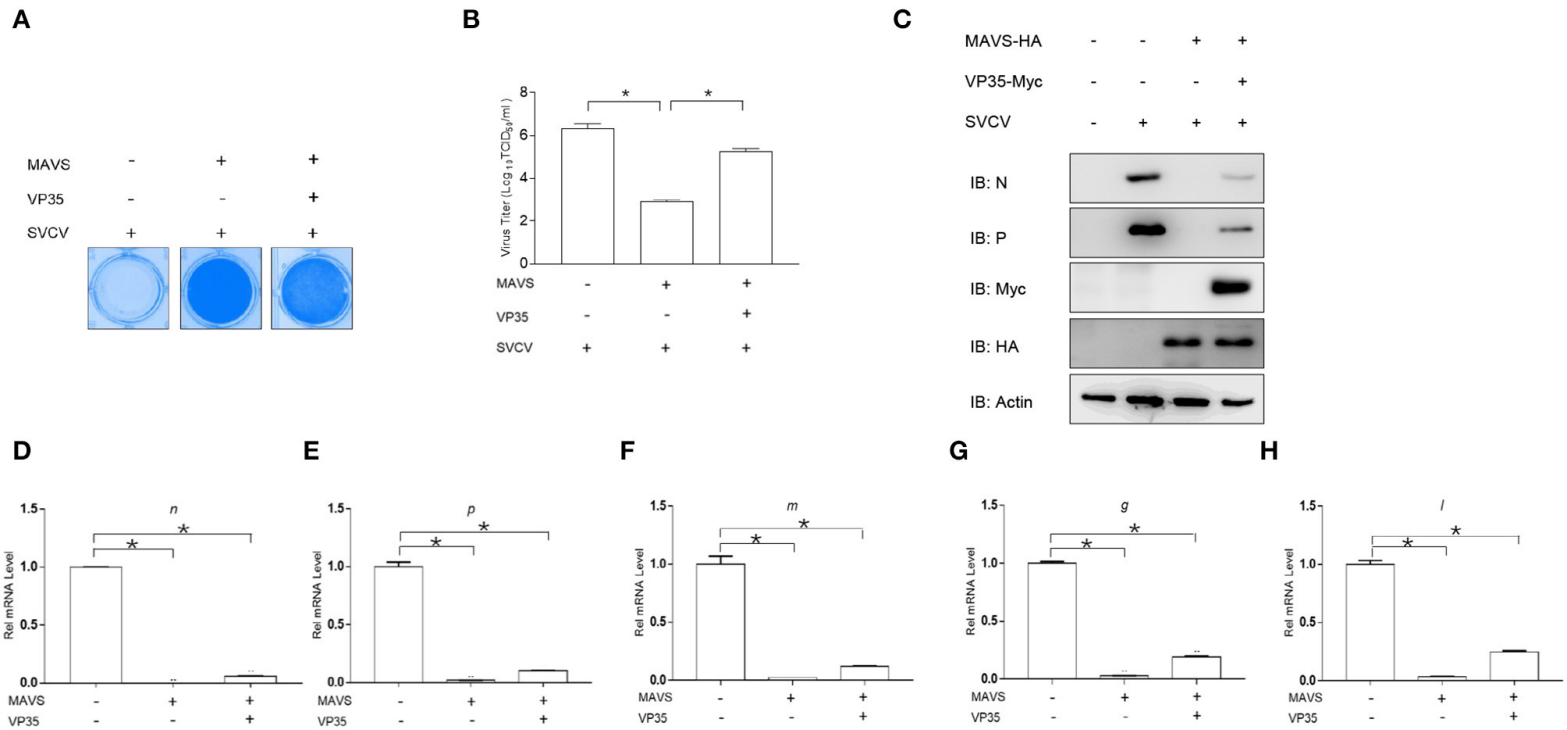
VP35 blocks MAVS-induced cellular antiviral immune response. **(A)** EPC cells seeded in 24-well plates overnight were transfected with 0.5 μg of MAVS-HA and 0.5 μg VP35-pcDNA3.1(+) or pcDNA3.1(+). At 24 h post-transfection, cells were infected with SVCV (MOI = 0.001) for 48 h. Then, cells were fixed with 4% paraformaldehyde (PFA) and stained with 1% crystal violet. **(B)** Culture supernatants from the cells infected with SVCV were collected, and the viral titer was measured according to the method of Reed and Muench. **(C)** EPC was seeded in six-well plates and transfected with 2 μg of MAVS-HA and 2 μg of VP35-Myc or empty vector. At 24 h post-transfection, cells were infected with SVCV (MOI = 1). After 24-h infection, the WCLs were detected by IB with the anti-N, anti-P, anti-Myc, anti-HA, and anti-β-actin Abs, respectively. **(D–H)** The same samples were prepared similarly as described above for **(C)**. Total RNAs were extracted to examine the mRNA levels of cellular *n, p, m, g*, and *l*. The relative transcriptional levels were normalized to the transcriptional level of the *β-actin* gene and were represented as fold induction relative to the transcriptional level in the control cells, which was set to 1. Data are expressed as mean ± SEM, *n* = 3. Asterisks indicate significant differences from control values (*p < 0.05).

Previous studies have reported that MAVS plays a crucial role in activating IFN expression and defending against DNA and RNA virus infections (29). In addition, the above results showed that VP35 targeted MAVS for the degradation. Thus, it is necessary to explore whether VP35 affects the antiviral function of MAVS. As shown in Figure 6A, upon infection with SVCV, the cells showed an obvious CPE. The overexpression of MAVS significantly protected the cells from virus infection, but VP35 weakened the protective effect of MAVS on cells remarkably. Then, we measured virus titers and found that the virus titer decreased 3,388-fold in MAVS-over-expressed cells compared to that in control cells, while the over-expressed VP35 attenuated the decrease in a virus titer caused by MAVS (Figure 6B). In addition, the qPCR and Western blot assays revealed that MAVS markedly inhibited the expression of viral genes at the mRNA and protein level, whereas VP35 weakened this effect (Figures 6C–H). These data suggest that VP35 negatively regulates the MAVS-mediated antiviral response.

### VP35 Reduces the Host Interferon Response and Promotes Virus Proliferation

**Figure 7:**
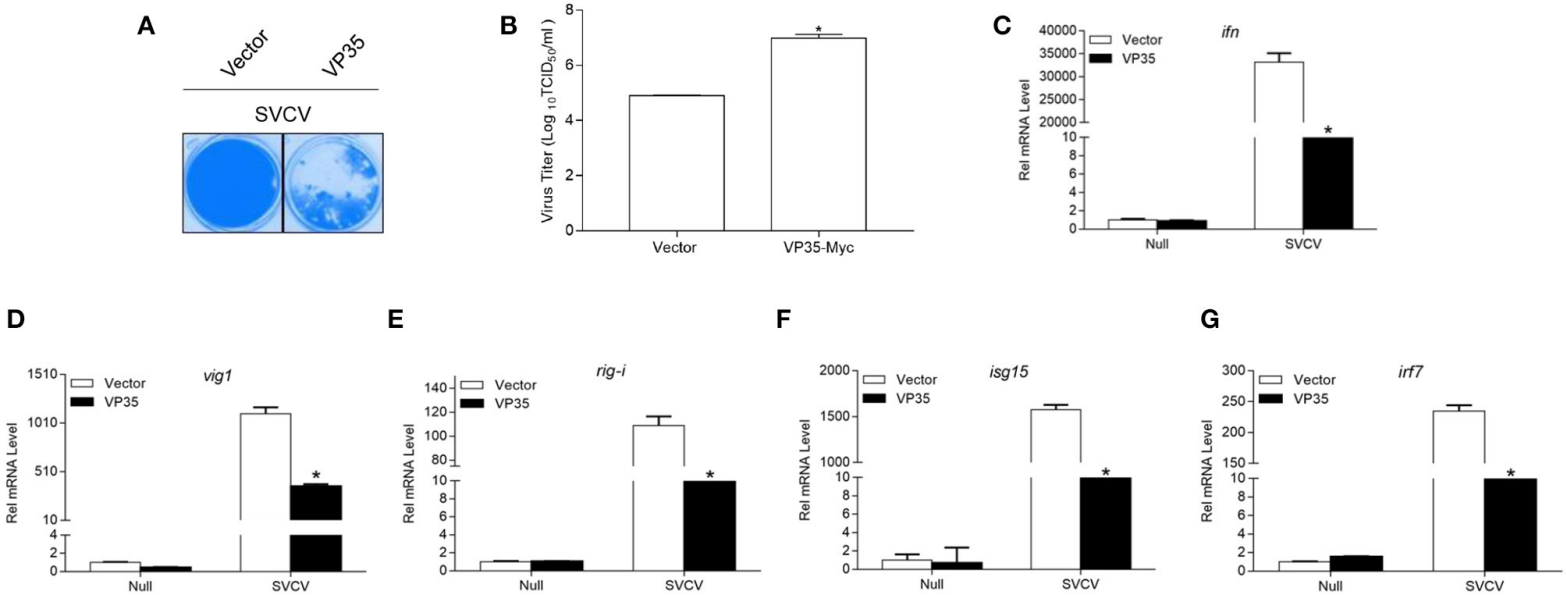
VP35 reduces host IFN response and promotes virus proliferation. **(A,B)** EPC cells seeded in 24-well plates overnight were transfected with 0.5 μg VP35-pcDNA3.1 (+) or pcDNA3.1 (+) vector. At 24 h post-transfection, cells were infected with SVCV (MOI = 0.001) for 48 h. **(A)** Then, cells were fixed with 4% PFA and stained with 1% crystal violet. **(B)** Culture supernatants from the cells infected with SVCV were collected, and the viral titer was measured according to the method of Reed and Muench. **(C–G)** EPC cells seeded into six-well plates overnight were infected with SVCV (MOI = 1). After 24 h, total RNAs were extracted to examine the transcriptional levels of cellular *ifn* **(C)**, *vig1* **(D)**, *rig-i* **(E)**, *isg15* **(F)**, *irf7* **(G)** by qPCR. The relative transcription levels were normalized to the transcription level of the *β-actin* gene and are represented as fold induction relative to the transcription level in control cells, which was set to 1. Data are expressed as mean ± SEM, *n* = 3. Asterisks indicate significant differences from control (*p < 0.05).

A determination is made whether VP35 affects the cellular IFN response to facilitate viral replication, and the EPC cells were transfected with VP35-Myc or the empty vector and infected with SVCV. As shown in Figure 7A, at 48 h post-infection, more CPE was observed in the VP35 group than in the control group. The measurement of the viral titer showed that the overexpression of VP35 increased the viral titer 120-fold compared to that in control cells (Figure 7B). In qPCR assays, SVCV infection upregulated the mRNAs of host *ifn, vig1, isg15-1, rig-i*, and *irf7* while the overexpression of VP35 significantly decreased these inductions (Figures 7C–G). Taken together, these results indicate that VP35 interferes with the host IFN response and facilitates the proliferation of SVCV.

## Discussion

Interferons are a class of cytokines with antiviral, antitumor, and immune regulation functions (37). IFNs do not directly kill viruses, but by secreting to the outside of the cell, binding to the intracellular IFN receptor, and activating a series of signaling pathways, they finally induce ISGs expression to clear virus (17, 38). Therefore, for viruses to replicate and proliferate in the body, they must find ways to disrupt host IFN system. For example, NS3 and NS2B3 of ZIKV negatively regulate the production of IFN by targeting MAVS and MITA for K48-linked ubiquitin–proteasome degradation (39). The fish IFN system also exhibits a powerful antiviral function. Meanwhile, aquatic viruses have evolved an enormous variety of strategies to circumvent the host immune response. The aquatic virus GCRV causes high mortality in grass carp; however, its immune escape mechanisms are not fully elucidated. Here, we demonstrated that GCRV VP35 interacts with and degrades MAVS *via* the autophagy pathway, thereby resulting in the host IFN inhibition. This finding has enriched the research on the immune escape mechanisms of aquatic viruses.

The adaptor protein MAVS, which links RIG-I to TBK1, plays a central role in inducing IFN expression in response to viral infection (40). Consequently, MAVS is targeted by many viruses to achieve immune escape. Various viral proteins have developed multiple strategies to disrupt MAVS-mediated signal transduction, such as degrading MAVS, cleaving MAVS, physically associating with MAVS, and breaking the function of MAVS-containing complexes (7, 41, 42). For example, rotavirus VP3 localizes at the mitochondria and induces the degradation of MAVS, blocking the production of IFN (43). Golgi protein 73 (GP73) of hepatitis C virus (HCV) acts as a negative regulator of the host immune response by interacting with MAVS/tumor necrosis factor (TNF) receptor-associated factor 6 (TRAF6) and facilitating their degradation (44). In this study, GCRV VP35 degrades MAVS through the autophagy pathway, suggesting a new strategy for the antagonism of MAVS by aquatic viruses. However, the specific autophagy mechanism involved in VP35 degrading MAVS requires further in-depth study.

Under normal circumstances of hosts getting infected with viruses, “contests” between the host immune system and viral proteins emerge in many forms. Since genomes of RNA viruses are generally small, the encoding proteins are limited. A common strategy is that various proteins encoded by one virus synergistically act on the same critical signaling pathway. For SVCV, the P protein competitively inhibits the kinase activity of TBK1, and the N protein degrades MAVS in a ubiquitin–proteasome manner, ultimately subverting the RLR signaling pathway (32, 45). For GCRV, our previous studies have revealed that VP41 targets MITA, and VP56 degrades phosphorylated IRF7 (46, 47). Our study has demonstrated that another protein of GCRV VP35 also acts on the RLR signaling pathway. The other strategy of virus is to use one protein to antagonize multi-host signaling pathways. For instance, the 3C protease (3C^pro^) of Seneca Valley virus (SVV) cleaves MAVS, Toll/interleukin 1 (IL-1) receptor domain-containing adaptor inducing IFN-β (TRIF), and TANK, resulting in the suppression of the RLR/Toll-like receptor 3- (TLR3-) mediated signaling pathway (48). Hence, further study is required whether other proteins encoded by GCRV modulate the RLR signaling pathway and these reported proteins negatively regulate other signaling pathways.

In fact, in the battle between the host and virus, several host molecules have been identified to counter viral proteins, making the suppression of viral immune evasion. For instance, IFN-inducible GTPase 1 (IIGP1) interacts with rabies virus (RABV) phosphoprotein and impedes its dimerization to restrict viral replication. IIGP1 impedes the dimerization of RABV 1 phosphoprotein and restricts viral replication Viperin (virus inhibitory protein), which is known as an ISG and possesses significant antiviral capacity, is induced during RABV infection and to reduce cholesterol and sphingomyelin in the cellular membrane to defend against virus proliferation. Viperin inhibits the RABV replication *via* reduced cholesterol and sphingomyelin and is regulated upstream by TLR4. The tumor suppressor p53 enhances IFN-induced TM protein 3 (IFITM3) palmitoylation, which is mediated by zinc finger DHHC domain-containing protein 1 (ZDHHC1) to decline Japanese encephalitis virus (JEV) replication. p53 promotes ZDHHC1-mediated IFITM3 palmitoylation to inhibit JEV replication. These studies demonstrate that multiple mechanisms of host exist in antiviral infection, meaning that the combat between the host and virus is more complicated than we know.

In summary, our study uncovers detailed mechanisms for the GCRV VP35-mediated immune evasion, which interacts with and promotes MAVS degradation in an autophagy manner. These findings reveal a novel immune escape mechanism used by GCRV and may provide new insights into the pathogenesis driven by GCRV–host interactions.

## Data Availability Statement

The raw data supporting the conclusions of this article will be made available by the authors, without undue reservation.

## Author Contributions

SL conceived and designed the experiments. SL, L-FL, CZ, Z-CL, X-YZ, J-YJ, DC, and Y-AZ performed the experiments and analyzed the data. SL, L-FL, and CZ wrote the manuscript. All authors reviewed the manuscript.

## Conflict of Interest

The authors declare that the research was conducted in the absence of any commercial or financial relationships that could be construed as a potential conflict of interest.
